# Agricultural total factor productivity, digital economy and agricultural high-quality development

**DOI:** 10.1371/journal.pone.0292001

**Published:** 2023-10-04

**Authors:** Dandan Gao, Xiaogang Lyu

**Affiliations:** Party School of Liaoning Provincial Party Committee, Shenyang, China; University of Galway, Ireland / Anhui University of Finance and Economics, CHINA

## Abstract

The long-term and stable development of agriculture is the key to China’s economic development and social stability. Agricultural total factor productivity and the digital economy have become new kinetic energy and new engines driving agricultural high-quality development. It is of great significance to verify whether there are significant spatial and threshold effects in the process of high-quality development of agriculture and to explore the intrinsic relationship between high-quality development of agriculture and agricultural total factor productivity and digital economy. This paper takes 31 provinces in China from 2011 to 2020 as the research object. The coefficient of variation method is used to estimate the comprehensive evaluation index of agricultural high-quality development and digital economy. And Dea-Malmquist index method is used to estimate agricultural total factor productivity. On this basis, the spatial Durbin model and threshold regression model are constructed to explore the spatial and threshold effects of agricultural total factor productivity, digital economy and other factors and high-quality agricultural development. The conclusion is as follows: the high-quality development of agriculture has significant spatial autocorrelation. Agricultural total factor productivity and digital economy have significant direct effect and indirect spillover effect on the high-quality development of agriculture. Agricultural total factor productivity has stage differences in each range of digital economy level, but its influence on agricultural high-quality development shows a positive state. Based on this, the paper puts forward some countermeasures to promote the high-quality development of agriculture.

## 1 Introduction

China is a large agricultural country, and the report of the 20th Party Congress clearly points out that we should adhere to the priority development of agriculture and rural areas and accelerate the building of a strong agricultural country. From the China Rural Statistical Yearbook, it can be found that the average growth rate of the total output value of agriculture, forestry, animal husbandry and fishery from 2000 to 2011 was 11.04%, while the average growth rate of the total output value of agriculture, forestry, animal husbandry and fishery from 2011 to 2021 has become significantly slower, with its average growth rate being 6.43%, which shows that China’s agricultural development has entered a critical period of gear shifting and quality improvement, and the main direction of the current agricultural development is to accelerate the transformation of the agricultural development model and further improve the quality of agricultural development. The report of the 19th CPC National Congress states that “raising total factor productivity is an important and dependable path to achieving high-quality economic development”. Total factor productivity is an important tool for measuring the quality of economic growth and clarifying the driving force of economic growth. It can reflect the economic growth brought by other factors after excluding factor inputs [1,2]. Long SB, Zhang MX (2021) and other scholars have proposed to promote the efficiency of the agricultural supply system through “green agriculture”, “quality agriculture” is an important path to promoting high-quality development of agriculture [3]. The national “14th Five-Year Plan” puts forward accelerating the development of the digital economy is a realistic need to build a new engine of high-quality development. With the rapid development of big data, the Internet of Things and other new-generation information technology, the digital economy has continuously empowered agriculture, and become a new driving force and a new engine for high-quality development of agriculture [4–6]. Zhou QX, Li XE (2022) and other scholars proposed that the digital economy can break the spatial and temporal constraints of information exchange, effectively promote the sharing of agricultural production information, resource integration and interconnection of factors, thereby enhancing the matching efficiency of the factors of agricultural production, promoting the transformation of the old and new kinetic energy, and boosting the high-quality development of agriculture [7,8]. Therefore, it is of great theoretical significance and practical value to explore the relationship between agricultural total factor productivity, digital economy and agricultural high-quality development.

At present, scholars at home and abroad have provided clearer and more accurate descriptions of the conceptual connotations, influencing factors and research methods of agricultural total factor productivity, digital economy and high-quality development of agriculture. For high-quality agricultural development, scholars have used subjective and objective assignment methods, multi-objective function method, Dagum Gini coefficient, threshold regression model and spatial econometric model to measure and decompose high-quality agricultural development from different perspectives [9–11], and clarified that the factors such as trade opening, China-ASEAN free trade zone tariff concessions for intermediate goods, the construction of the two types of society, agricultural socialization services, digital inclusive finance, green financial support, agricultural technology progress, agricultural science, technology innovation, digital economy, land transfer policy, agricultural tourism integration, rural infrastructure, agricultural productive services, labor migration, and population aging have contributed to or inhibiting effects [12–24]. For total factor productivity in agriculture, scholars have used SFA (stochastic frontier model), DEA (data envelopment analysis model) and Cobb-Douglas function to measure and decompose it, and clarify the relationship between factors such as capital deepening, digital countryside, agricultural insurance, agricultural trade liberalization and total factor productivity in agriculture [25–28]. For the digital economy, scholars have constructed a comprehensive evaluation index system from multiple perspectives, examined its spatial and temporal characteristics as well as regional differences, and explored its empowering effects. However, the relationship between total factor productivity in agriculture, the digital economy and high-quality agricultural development has been less explored, and mainly between the two of them. The research on digital economy and agricultural high-quality development mainly includes: first, exploring the relationship between digital economy and agricultural high-quality development. Li BQ (2022), Lu ZY (2022) and other scholars based on the role of the digital economy empowered agricultural high-quality development mechanism for empirical testing, found that the digital economy on agricultural high-quality development has a nonlinear positive impact, and has regional heterogeneity [29,30]. Second, the practice path of digital economy empowered agricultural high-quality development. Luo QF, Zhao QF (2022) have proposed that to promote the high-quality development of agriculture, we should strengthen the positive external effects of digital technology standards and technological innovation, improve the level of digital services and the ability to use digital technology, and play the role of digital technology in promoting the integration of agricultural development and the expansion of functions [6]. Zhou QX, Li XE (2022) proposed to vigorously promote the construction of digital infrastructure as a breakthrough, the development of digital technology as a focus point, tapping the potential for endogenous development of agriculture, expanding the digital application scenarios, promoting the deep integration of the digital economy and rural agriculture, creating a distinctive brand of agricultural products, and promoting the high-quality development of agriculture [8]. For the study of the digital economy and agricultural total factor productivity: Sun GL (2023), Lin QN (2022) and other scholars found that the digital economy has a significant positive impact on agricultural total factor productivity, and regional differences are obvious [31,32]. For the study of agricultural total factor productivity and agricultural high-quality development: scholars such as Long SB, Zhang MX (2021) used agricultural total factor productivity to measure the level of agricultural high-quality development [3]. Xu PJ (2019) and Wu G (2022) proposed that total factor productivity improvement has a significant positive impact effect on high-quality development [33,34]. The above studies provide useful references and reference for understanding the relationship between agricultural total factor productivity, digital economy and agricultural high-quality development. However, there are certain limitations in the existing literature:(1) The current research on the interaction relationship between agricultural total factor productivity, digital economy and agricultural high-quality development is still weak, and moreover, there is a lack of detailed discussion on its spatial effect and threshold effect. (2) With regard to the indicators for measuring the digital economy and the high-quality development of agriculture, different scholars do not select the indicators in a uniform manner, and some of the indicators are not selected reasonably enough. Can agricultural total factor productivity and digital economy drive agricultural high-quality development? What is the mechanism between the three? In order to answer the above questions, this paper, on the basis of existing research, verifies whether there is spatial correlation and agglomeration of agricultural high-quality development, and establishes spatial regression model and threshold regression model to explore the direct effect, spatial spillover effect and threshold effect of agricultural total factor productivity and digital economy on agricultural high-quality development, with a view to providing certain reference for the road of agricultural high-quality development in China.

## 2. Theoretical analysis and research hypothesis

### 2.1 The impact of agricultural total factor productivity on high-quality agricultural development

Total factor productivity in agriculture mainly affects the high-quality development of agriculture through technological progress and efficiency improvement. Technological progress in agriculture is mainly manifested in the improvement of agricultural management level and the improvement of agricultural innovation capacity. This can improve the working skills of farmers, improve or create more advanced agricultural production tools, which in turn can improve the quality and structure of agricultural factors and promote the process of division of labor and specialization in agriculture. Improvements in agricultural efficiency are mainly in the form of increases in pure technical efficiency and scale efficiency. The increase in agricultural efficiency allows agriculture to unleash greater potential by increasing the coordination between various resource factors under the current level of technology, thus achieving greater overall efficiency per unit of time. Agricultural production factors, enterprises and sectors continue to converge to higher value-added areas due to the price transmission mechanism, creating scale effects and industrial agglomeration.

H1: Total factor productivity in agriculture has a significant positive effect on high-quality agricultural development.

### 2.2 The impact of the digital economy on the high-quality development of agriculture

The digital economy mainly influences the high-quality development of agriculture through the accumulation and sharing of data elements and the innovation and application of digital technology. The accumulation and sharing of data elements can enable farmers, producers and processors, the government and other relevant interest groups to grasp the basic situation of agriculture and rural areas, relevant government policies, as well as a large number of information resources such as supply and demand, weather conditions, pests and disasters, etc., create good conditions for communication between township governments and residents, reduce the transaction costs of obtaining information, break down barriers to information exchange, increase the circulation of data and information and knowledge elements speed, reduce the mismatch between supply and demand and ineffective supply caused by information asymmetry and information lag, realize the interoperability and linkage of agricultural production, circulation and consumption, and improve production efficiency. The innovation and application of digital technology can provide real-time monitoring of the data required in agricultural production, provide efficient, accurate and intelligent technical support, and enhance digital and smart agriculture. At the same time, it also changes the traditional way of trading in agriculture, strengthening the connection between different subjects through online platforms and live streaming software, reconfiguring the production supply chain, releasing and reducing redundant links, broadening the sales channels and scope, and expanding the scale of cultivation and production.

H2: The digital economy has a significant positive effect on the high-quality development of agriculture.

### 2.3 Spatial spillover and threshold effects of high-quality agricultural development

Total factor productivity in agriculture and the digital economy are shared, permeable, external and diffuse, and can break through spatial restrictions and constraints to achieve the flow of information, factors and technology. Neighboring regions can enhance the breadth and depth of their own agricultural activities by absorbing advanced agricultural technologies, experiences and outstanding achievements from advanced regions. The fragmented state of agriculture-related industries is broken, and planting, breeding, tourism, services, catering, transportation, and logistics are closely linked to promoting the integrated development of various regions and industries.

The digital economy has an important impact on total factor productivity in agriculture, and existing research shows that technological innovation can drive technological progress and efficiency improvements in agriculture, thereby increasing total factor productivity. At the same time, the development of the digital economy can break the constraints of time and space on the dissemination of agricultural knowledge, information and technology, accelerate the integration and optimization of factors, improve the efficiency of resource allocation, and thus optimize agricultural total factor productivity. The impact of the digital economy on agricultural total factor productivity is marginal, and when the level of the digital economy is not high, the impact of agricultural total factor productivity on high-quality agricultural development may be overshadowed by other factors, so that it cannot fully play its role in promoting high-quality agricultural development. That is, the enhancing effect of agricultural total factor productivity on high-quality agricultural development is influenced by the intensity of the digital economy and may show a non-linear relationship.

H3: Total factor productivity in agriculture and the digital economy have spatial spillover and have a significant positive effect on the high-quality agricultural development of the surrounding areas.
H4: The influence of agricultural total factor productivity on the high-quality development of agriculture has the threshold effect of digital economy.

## 3 Research methodology and indicator system construction

### 3.1 Research methodology

#### 3.1.1 Coefficient of variation method

In this paper, the coefficient of variation method is chosen to measure the comprehensive evaluation index of high-quality agricultural development and digital economy. The calculation steps of this method are [35]:

Standardized initial data:                $x_{ij}=\frac{x_{ij}-min(x_{ij})}{\max\left(x_{ij}\right)-min(x_{ij})}$
Mean:                           $\bar{x_{j}}=\frac{1}{n}\sum_{i=1}^{n}x_{ij}$
Standard deviation:                    $s_{j}=\sqrt{\frac{\sum_{i=1}^{n}{\left(x_{ij-}\bar{x_{j}}\right)}^{2}}{n-1}}$
Coefficient of variation:                    $v_{j}=\frac{s_{j}}{\bar{x_{j}}}$
Weight:                                $A_{j}=\frac{v_{j}}{\sum_{j=1}^{n}v_{j}}$

#### 3.1.2 Dea-Malmquist index method

In this paper, the DEA-Malmquist index method is chosen to measure total factor productivity in agriculture. It enables the calculation of dynamic rates of change in adjacent periods and allows further decomposition of total factor productivity, which helps to explore the drivers of change in depth. The formula for this method is [36]:

TFP=EFFCH*TECHCH=PECH*SECH*TECHCH

#### 3.1.3 Moran’s index

In this paper, the Moran index is used to explore whether high-quality agricultural development is spatially relevant. The index is calculated as [37]:

Global Moran index:

$$I=\frac{n\sum_{i=1}^{n}\sum_{j=1}^{n}w_{ij}(x_{i}-\bar{x})(x_{j}-\bar{x})}{\sum_{i=1}^{n}\sum_{j=1}^{n}w_{ij}\sum_{i=1}^{n}{\left(x_{i}-\bar{x}\right)}^{2}}$$

Where *n* is the number of regions, *w*_*ij*_ is the spatial weight matrix of regions *i* and *j*, and *x* is the target variable (level of quality agricultural development). When I passes the test of significance level, I>0 means positive correlation; I<0 means negative correlation; I = 0 means no spatial correlation exists.

#### 3.1.4 Spatial panel regression model

In judging and selecting the optimal spatial econometric model, firstly, the overall model fitting effect (R^2^, T-value, etc.) should be judged; secondly, the choice of fixed effects or random effects (Hausman test—Hausman test) should be judged; finally, the existence of spatial lags, spatial errors, and whether the spatial Durbin model can degenerate into a spatial autocorrelation or spatial error model (Lagrange multiplier-LM, robust Lagrange multiplier-R-LM, likelihood ratio-LR and Wald test-Wald test). The spatial panel model expression is [38]:

Y=Xβ+WXδ+ρWY+μ+v+ξ

ξ=λWξ+ε

Where Y is the level of quality agricultural development (vector of dependent variables), X is total factor productivity in agriculture, digital economy, urbanization, infrastructure development, and government intervention (matrix of independent variables), W is a matrix of spatial weights, μ and v are fixed and random effects, is a vector of residuals, β is a non-spatial term coefficient, δ, ρ and λ are spatial term coefficients, and ε is a vector of white noise.

When λ = 0,ρ≠0,δ = 0, it is a spatial autoregressive model; when λ≠0,ρ = 0,δ = 0, it is a spatial error model; when λ = 0,ρ≠0,δ≠0, it is a spatial Durbin model.

#### 3.1.5 Threshold regression model

In this paper, the Bootstrap method (Hansen, 1999) was used to draw samples 300 times for significance testing of the threshold effect and Wang Qunyong’s threshold regression model instruction (xthreg) to establish a threshold regression model to determine whether there is a threshold effect, as well as the number of thresholds and threshold values. When it is determined that there is a single threshold effect, the existence of double thresholds as well as multiple thresholds can be further explored.

The expression for the threshold regression model is (single threshold model) [39]:

$$y_{it}=\beta_{1}x_{it}I(q_{it}<\gamma )+\beta_{2}x_{it}I(q_{it}\geq \gamma )+\varepsilon_{it}+\mu_{i}$$

Where *y* is the explanatory variable, *x* is the explanatory variable, i is the region, t is the year, β is the coefficient vector, ε is the error vector, *I* is the indicator function, *q* is the threshold variable, γ is the threshold value and μ is the individual effect.

### 3.2 Indicator system construction

At present, the academic community has not yet formed a comprehensive evaluation index system for high-quality development of agriculture and digital economy. In this paper, on the basis of following the principles of systematicity, scientificity and comprehensiveness, a combination of theoretical analysis, frequency analysis and expert consultation is adopted for the selection of indicators for high-quality development of agriculture and the digital economy. Drawing specifically on the research results of Yang N (2022), Liu ZY (2021), Ma XJ (2022), Gao DD (2023) and Yin CJ (2022) [40–44], this paper seeks to comprehensively and accurately reflect the reality of high-quality agricultural development and digital economy in China, as shown in Tables 1 and 2. The measurement of total factor productivity in agriculture is relatively mature in academia, with input variables including labor (the number of employees in the primary industry), land (the area of crops sown), fertilizer (the discounted amount of fertilizer applied to agriculture) and capital (the total power of agricultural machinery), and output variables being the value added of agriculture, forestry, animal husbandry and fishery.

**Table 1:** Comprehensive evaluation index system for high-quality agricultural development.

Target layer	Criterion layer	Indicator layer	Unit	Attribute
		Ratio of added value of agriculture, forestry, husbandry and fishery to GDP	%	Positive
		Growth rate of added value of agriculture, forestry, husbandry and fisheries	%	Positive
	Economic vitality	Personal investment in fixed assets completed in agriculture	Ten thousand yuan	Positive
		Agricultural labor productivity	%	Positive
		Land yield rate	%	Positive
		Index of crop diversification	%	Positive
		Meat product diversity index	%	Positive
	Structural optimization	Expenditure on agriculture, forestry and water conservancy as a proportion of total government expenditure	%	Positive
		Industrial restructuring index	%	Positive
		Bivariate correlation coefficient	%	Positive
		Fertilizer application intensity	Ton/ha	Negative
		Pesticide application intensity	Ton/ha	Negative
High-quality development of agriculture	Green ecology	Agricultural energy intensity	Ton/ha	Negative
		Service strength of agricultural plastic film	Ton/ha	Negative
		Area intensity of soil erosion control	%	Positive
		Growth rate of agricultural exports	%	Positive
	Open innovation	Agricultural externality index	%	Positive
		Agricultural mechanization level	Kw/ha	Positive
		Per capita rural power generation	KWH	Positive
		Per capita effective irrigation area	Ha.	Positive
		Rural- urban income ratio	%	Positive
	Harmonious sharing	Growth rate of per capita annual income of rural households	%	Positive
		Engel coefficient	%	Negative
		Rural doctors and health workers per 1,000 agricultural population	Person	Positive
		Growth rate of rural cultural station	%	Positive

**Table 2:** Comprehensive evaluation indicator system for the digital economy.

Target layer	Criterion layer	Indicator layer	Unit	Attribute
		Length of fiber optic cable lines per square kilometer	Km	Positive
		Number of Internet broadband access ports per square kilometer	A	Positive
	Digital Foundations	Number of domain names per 100 people	A	Positive
		Number of web pages per 100 people	A	Positive
		Main business income of electronic information manufacturing industry as a proportion of GDP	%	Positive
		Software business revenue as a percentage of GDP	%	Positive
	Digital Industries	Proportion of employed persons in urban units of information transmission, software and information technology service industry to total employed persons	%	Positive
		Share of employment in the electronics and communications equipment manufacturing industry in total employment	%	Positive
Digital Economy		Internet broadband access users per 100 population	a / 100 people	Positive
		Mobile phone penetration rate	a / 100 people	Positive
	Digital Applications	Share of enterprises with e-commerce transaction activities	%	Positive
		Share of e-commerce sales in GDP	%	Positive
		Number of computers used per 100 people	a / 100 people	Positive
		Full-time equivalent of R&D personnel in industrial enterprises above designated size	Person-years	Positive
		R&D expenditure of industrial enterprises above scale	Ten thousand yuan	Positive
	Digital Innovation	Number of patent applications for inventions by industrial enterprises above designated size/number of patent applications by industrial enterprises above designated size	%	Positive
		Share of technology market turnover in GDP	%	Positive

### 3.3 Data sources and description of variables

The data in this paper were obtained from the *China Statistical Yearbook*, *China Environmental Statistical Yearbook* and *China Rural Statistical Yearbook* from 2010 to 2020. For individual missing data, the smoothing index method, linear interpolation method and moving average method were used to supplement them.

In the spatial panel regression model, the dependent variable, the level of high-quality development in agriculture, is represented by (Y). The independent variables are total factor productivity (TFP) and digital economy (DIG) in agriculture. The control variables are urbanization (URB), infrastructure development (INF) and government intervention (FIS). In the threshold regression model, the dependent variable is quality agricultural development (Y), the independent variable is total factor productivity in agriculture (TFP), the threshold variable is the digital economy, and urbanization (URB), infrastructure development (INF) and government intervention (FIS) are the control variables. Where total factor productivity in agriculture is measured using the Malmquist index and is cumulative.

## 4 Empirical analysis

### 4.1 Analysis of the spatial effect of high-quality development of Chinese agriculture

According to the results of the global Moran index (Table 3), it can be seen that the Moran index is greater than 0 and passes the significance test. It indicates that there is a significant spatial dependence of the level of agricultural high-quality development in space, and therefore a spatial factor should be introduced when building the regression model. According to the results of the LR and Wald tests (Table 4), the LR-SAR, LR-SEM, Wald-SAR and Wald-SEM tests all passed the 1% significance test, indicating that the spatial Durbin model cannot be reduced to a spatial error model or a spatial lag model, and the choice of the spatial Durbin model to explore the spatial spillover effects of total factor productivity in agriculture and the digital economy on the level of high-quality development in agriculture is The R^2^ was 0.9472, indicating that the spatial Durbin model fits well. According to the results of the Hausman test, the fixed effects of the spatial Durbin model are all better than the random effects, so the analysis should be based on fixed effects.

**Table 3:** Global Moran index from 2011 to 2021.

	I	P
2011	0.4920	0.001
2012	0.4904	0.001
2013	0.4584	0.001
2014	0.4287	0.001
2015	0.4377	0.001
2016	0.4118	0.002
2017	0.3228	0.009
2018	0.3480	0.005
2019	0.2714	0.007
2020	0.3768	0.003

**Table 4:** Regression results of SDM.

Variable	Coefficient	Variable	Coefficient
TFP	0.0313*** (8.6524)	W*TFP	0.0203*** (2.9933)
DIG	0.2739*** (9.7247)	W*DIG	0.0137 (0.2840)
URB	-0.1850*** (-3.5432)	W*URB	-0.1155 (-1.4818)
INF	0.0623 (0.6351)	W*INF	0.4208** (1.8919)
FIS	0.0388 (1.3012)	W*FIS	0.1257* (2.4243)
W*Y	0.3550*** (5.2890)	LR-SAR	14.8953**
R^2^	0.9472	LR-SEM	44.9246***
Hausman text	50.2754***	Wald-SAR	13.7887**
log likelihood	990.169	Wald-SEM	38.4806***

Note: ***, **, * represent statistical significance at the level of 1%, 5% and 10% respectively.

According to the results of the spatial Durbin model (Table 4), the spatial autoregressive coefficient of the level of high-quality agricultural development was 0.3550, which passed the 1% significance test, which was consistent with the test results of the spatial association effect. The regression coefficients of agricultural total factor productivity and digital economy were 0.0313 and 0.2739 respectively, indicating a positive correlation between agricultural total factor productivity, digital economy and high-quality agricultural development, and all of them passed the 1% significance test. From the spatial lagged variables, we can see that the coefficient of the spatial interaction term of agricultural total factor productivity passed the 1% significance test, and the regression coefficient of 0.0203, is positively correlated with the high-quality development of agriculture. It verifies that the high-quality development of agriculture is open and there are external spillover effects.

As the spatial Durbin model contains a spatial lag term for the variables, the regression results generated by the run cannot accurately estimate the impact of the explanatory variables on the region and the explanatory variables in neighboring regions [37]. Therefore, the model further decomposes the marginal effects into direct effects (local effects) and indirect effects (spillover effects), as shown in Table 5.

**Table 5:** Decomposition of marginal effect of SDM.

Variable	Direct Effect	Indirect Effect	Total Effect
TFP	0.0343*** (9.5969)	0.0460*** (5.9299)	0.0803*** (9.1767)
DIG	0.2837*** (9.7525)	0.1629** (2.6692)	0.4465*** (6.5834)
URB	-0.2043*** (-4.2252)	-0.2663*** (-2.7608)	-0.4707*** (-5.0565)
INF	0.0987 (0.9321)	0.6352* (1.8325)	0.7339* (1.7806)
FIS	0.0529* (1.8242)	0.2067*** (2.8811)	0.2596*** (3.4449)

Note: ***, **, * represent statistical significance at the level of 1%, 5% and 10% respectively.

The direct and indirect effects of agricultural total factor productivity on the level of quality agricultural development were 0.0343 and 0.0460 respectively, and both passed the 1% significance test. This implies that the improvement of agricultural total factor productivity has a significant contribution to the improvement of high-quality agricultural development. At the same time, the improvement of total factor productivity in agriculture in neighboring provinces will also drive the high-quality development of agriculture in the province. The direct and indirect effects of the digital economy on the level of high-quality agricultural development were 0.2837 and 0.1629 respectively, and both passed the 5% significance test. This implies that the enhancement of the digital economy has a significant contribution to the high-quality development of agriculture. At the same time, the enhancement of the digital economy in neighboring provinces will also drive the high-quality development of agriculture in the province. This is mainly because total factor productivity in agriculture, as an important indicator to measure the quality of agricultural development, its enhancement leads to improved agricultural management, continuous improvement of the system, improvement of the crude development method in the process of agricultural development. Agricultural technology is improved and innovated, increasing the added value of agricultural products, and the input and use of agricultural factors are reasonably allocated, which plays a role in improving quality and increasing efficiency. This in turn improves the level of quality development of local agriculture. The application of digital technology in agriculture has led to changes in agricultural production methods, promoting the optimization and upgrading of traditional input factor structures, improving factor allocation efficiency and continuously promoting the integration of old and new business models. At the same time, the increase in total factor productivity and digital economy in the province’s agriculture can spread to the periphery, allowing the “periphery” to absorb and learn from its management experience, operational models and advanced knowledge and technology, thereby driving high-quality agricultural development in neighboring provinces.

### 4.2 Analysis of threshold effects for high-quality development of Chinese agriculture

From the results of the threshold effect test (Table 6), the single threshold estimate of 0.41 passed the significance test at the 1% level, suggesting the existence of at least 1 threshold; the double threshold test did not pass the significance tests at 1%, 5% and 10%, suggesting the non-existence of a 2nd threshold. Therefore, there is a threshold of 1 for the level of quality development in agriculture. After determining the existence of a threshold effect and the number of thresholds, the single threshold was estimated and the results showed a threshold of 0.41 for the digital economy at the 95% confidence level. from the threshold regression model for high-quality development in agriculture in Table 7, it can be seen that urbanization and government intervention have a positive effect on high-quality development in agriculture. The non-linear positive impact of agricultural total factor productivity on the level of agricultural quality development, agricultural total factor productivity has a stage difference between the various zones of the digital economy level, but the impact on agricultural quality development shows a positive upward state. When the digital economy level (Y) is less than 0.41, the level of agricultural quality development increases by 0.0385 for each unit increase in agricultural total factor productivity; when Y≥0.41, the level of agricultural quality development increases by 0.0817 for each unit increase in agricultural total factor productivity. The effect of total factor productivity on the quality development of agriculture has a significant and gradually increasing effect.

**Table 6:** Testing the threshold effect of high-quality agricultural development.

Variable	Single threshold	Double threshold
Single threshold estimate	0.4099 (0.3927, 0.4172)	0.4099 (0.3927, 0.4172)
Double threshold estimate		0.2586 (0.2528, 0.2598)
F	102.82	30.50
P	0	0.2200

**Table 7:** Test of threshold effect.

Variable	Model 1
URB	0.0915** (2.5)
INF	0.0794 (0.64)
FIS	0.0659* (1.94)
Threshold coefficient 1	0.0385*** (9.1)
Threshold coefficient 2	0.0817*** (12.82)
Constant	0.1646*** (10.16)
R-squared	0.6663

Note: ***, **, * represent statistical significance at the level of 1%, 5% and 10% respectively.

In summary, all hypotheses proposed in this paper are valid, as shown in Table 8.

**Table 8:** Hypothesis induction summary.

Assumptions	Specific description	Conclusion
H1	Total factor productivity in agriculture has a significant positive effect on high-quality agricultural development.	Holds true
H2	The digital economy has a significant positive effect on the high-quality development of agriculture.	Holds true
H3	Total factor productivity in agriculture and the digital economy have spatial spillover and have a significant positive effect on the high-quality agricultural development of the surrounding areas.	Holds true
H4	The influence of agricultural total factor productivity on the high-quality development of agriculture has the threshold effect of digital economy.	Holds true

## 5 Conclusions and implications

Based on China’s provincial panel data from 2011–2020, this paper innovatively analyses the mechanism of agricultural total factor productivity and digital economy on agricultural high-quality development. Using Moran index, spatial Durbin model and threshold regression model to empirically examine the spatial spillover effect and non-linear relationship between agricultural total factor productivity and digital economy on agricultural high-quality development, the following conclusions are drawn:

(1) The process of agricultural high-quality development is not isolated, and there is a significant spatial dependence on agricultural high-quality development in space. This is consistent with the conclusions drawn by Qin XJ (2020) [45]. The improvement of agricultural total factor productivity and digital economy has a significant role in promoting the improvement of agricultural high-quality development. At the same time, the improvement of agricultural total factor productivity and digital economy in neighboring provinces will also drive the high-quality development of agriculture in this province. The conclusions of the scholars such as Lu ZY (2022), Chen YH (2022), Suan GL (2023) from the side to confirm the reliability of the research conclusions of this paper [18,30,31]. The findings of this study provide practical guidance for vigorously developing the digital economy, improving total factor productivity in agriculture, stimulating innovation in agriculture and rural areas, and strengthening exchanges and cooperation with neighboring provinces to promote the high-quality development of agriculture. (2) There is a significant threshold effect in the process of agricultural high-quality development. With the improvement of the level of the digital economy, the impact of agricultural total factor productivity on agricultural high-quality development has a significant upgrading effect, and the effect is gradually increasing. This indicates that the impact of factors at different threshold stages of high-quality development of agriculture varies, so it should be optimized and adjusted according to the needs of reality, so as to promote the level of high-quality development of agriculture. This conclusion further clarifies the key point and focus points for promoting the high-quality development of farmers through the development of the digital economy. Based on this, the following recommendations are made:

(1) Regions should pay sustained attention to the construction of rural digital infrastructure, expand the coverage of rural networks, break down information barriers and promote information exchange and collaborative operations among all parties. Through policy guidance, promote innovation and transformation of scientific research results in digital technology in agriculture, develop and promote digital information technology and agricultural production equipment adapted to high-quality agricultural development, and improve the level of agricultural science and technology innovation and intelligent agricultural mechanization. Strengthen professional training and policy support for digital skills and literacy of agricultural practitioners, attract technology enterprises and technical talents to join the team, create a high-level digital talent pool, and improve the suitability of agricultural labor endowment and digital technology.

(2) Each region will promote the construction of an agricultural science and technology innovation system and platform in accordance with the actual situation of agricultural development, and enhance the overall effectiveness of the agricultural innovation system. Strengthen the transformation and application of agricultural science and technology achievements, take the path of special agricultural innovation and promote agricultural technology progress. The government and relevant agricultural departments should do a good job of top-level design, formulate scientific and orderly agricultural business planning, comprehensively promote plans and actions to enhance the high quality and quality of agriculture, strengthen the collaborative capacity of agriculture, reasonably allocate agricultural innovation factor inputs and ensure the smooth operation of the agricultural innovation system, thereby enhancing the efficiency of agricultural technology.

(3) Regions should abandon the concept of “local protectionism and self-consistent development” and take the initiative to exchange and cooperate with “pioneering” regions to absorb advanced agricultural technology, management experience and models. We should give full play to the radiating effect of high-quality agricultural development and the spatial spillover effects of positive factors such as total factor productivity and the digital economy, promote the flow of resources and factors between provinces and regions, and encourage regions with a high level of high-quality agricultural development to give more support to regions with a low level of high-quality agricultural development, so as to form a new pattern of win-win cooperation and virtuous development.

There are still some issues to be explored in depth in this study: firstly, this paper uses the digital economy as a threshold variable to examine the non-linear relationship between agricultural total factor productivity and agricultural high-quality development caused by changes in its state variables. In the future, the institutional variable (policy support) can also be considered as a threshold variable to identify the impact of institutional heterogeneity on the improvement of agricultural high-quality development. Secondly, this paper takes the panel data of 31 provinces in China as the research basis to explore the spatial and threshold effects of agricultural total factor productivity, digital economy and agricultural high-quality development at the provincial level, and to grasp the relationship between the three as a whole. It is hoped that with the establishment and improvement of agricultural satellite accounts, small regions or microdata can be measured in the future, which will be the focus of subsequent research.
